# Compassionate Communication During Early Pregnancy Loss: Using Forum Theatre to Train Emergency Medicine Residents

**DOI:** 10.1002/aet2.70260

**Published:** 2026-09-27

**Authors:** Maria Brann, Jennifer J. Bute, Kelsey E. Binion, Emilio G. Robles, Andreia B. Alexander, Malia J. Moore

**Affiliations:** ^1^ Indiana University Indianapolis Indianapolis Indiana USA; ^2^ University of Texas at Arlington Arlington Texas USA; ^3^ Indiana University School of Medicine Indianapolis Indiana USA

## Abstract

**Background:**

Compassionate communication is a critical skill when caring for patients experiencing early pregnancy loss (EPL) in the emergency department; however, medical learners often have limited opportunities to practice this skill in a psychologically safe, low‐stakes environment before interacting with patients experiencing a loss in the clinical setting.

**Objective:**

The goal of this innovation was to develop a novel simulation experience using forum theatre techniques to enhance empathy, confidence in communicating about loss, and compassionate communication skills of emergency medicine (EM) residents when diagnosing an EPL, delivering the diagnosis, and discussing management options while providing emotional support.

**Methods:**

Using forum theatre (i.e., an experiential learning technique that uses interactive theatre for exploring and performing transformative actions), we developed an innovative training for first‐year (PGY‐1) and second‐year (PGY‐2) EM residents to practice compassionate communication strategies. Post‐surveys, conducted immediately after the intervention, assessed the training's effectiveness to enhance empathy, demonstrate compassionate communication techniques, and encourage personal reflection.

**Results:**

Twenty‐three PGY‐1 and eight PGY‐2 EM residents participated in separate training sessions. Survey responses indicated enhanced empathy and confidence, recognition of the importance of addressing patients' emotional needs, awareness of compassionate communication strategies through modeled examples, and reflection of individual communication behaviors.

**Conclusion:**

An innovative, simulation‐based training using forum theatre offers learners a structured opportunity to practice and refine compassionate communication strategies. By enhancing EM residents' empathy and confidence in communicating compassionately with patients experiencing EPL, this educational experience offers a novel way to address communication gaps in training and can be easily integrated into existing curricula.

## 1 Need for Innovation

Compassionate and clear communication is recognized as a fundamental component of Emergency Medicine (EM) training, and the Accreditation Council for Graduate Medical Education (ACGME) recognizes its importance by including interpersonal and communication skills among the core milestones residents must achieve for program completion [1]. Patient‐centered communication (PCC) (i.e., communication that forefronts patient preferences, needs, and values and invites patients to actively participate in their health care) [2] was added in 2026 as a skill eligible for assessment on the American Board of Emergency Medicine (ABEM) certifying exam [3]. This change elevates PCC from a recommended aspect of high quality patient care to a competency directly tied to board certification in EM. Therefore, providing medical learners with training in effective communication skills is increasingly important.

Residency programs vary widely in how training is structured [4], particularly the extent and quality of communication skills education provided to learners [2]. Training programs ultimately shape the quality of care provided. In their scoping review of compassionate clinical practices for early pregnancy loss (EPL) in emergency departments (EDs), Gregory et al. [5] found substantial variation in the quality of care patients received. Furthermore, patients have reported dissatisfaction with EPL care, resulting in heightened emotional distress. This distress is due, in part, to a lack of PCC and compassionate care from health care providers; however, providers report barriers, such as insufficient training in compassionate care, uncertainty, and time constraints that hinder their ability to deliver optimal care [5].

## 2 Background

Each year in the United States, approximately 900,000 patients visit EDs with concerns about a potential EPL [6]. Oftentimes, pregnant women elect to visit an ED instead of seeking care at an outpatient facility because of the urgency and uncertainty associated with their symptoms (e.g., bleeding after a confirmed pregnancy) [7, 8] or because of limited access to primary obstetric care [9]. In the ED, health care providers focus on ruling out ectopic pregnancy (i.e., a fetus develops outside the uterus), confirming nonviability, and medical management (e.g., medication, surgery) [10]. Although EDs are accessible for many pregnant patients, research shows that patients have reported negative experiences due to limited information, lack of emotional support, feeling their needs were not prioritized, and enduring long wait times [7, 11]. To improve patient experiences, research suggests that health care providers should offer expedited evaluation of EPL as well as clinical and emotional care of EPL [11]. Preparing health care providers to deliver this level of care requires training approaches that address both technical and interpersonal skills.

Medical learners have begun to recognize the value of using the arts and humanities, specifically theatre, to learn about complex topics like compassionate communication [12, 13, 14, 15]. Theatre techniques, such as facilitated improvisation and role‐play, allow learners the opportunity to practice compassionate communication techniques in a safe environment while exploring emotions and fostering empathy by receiving immediate feedback and reflecting on the experience [16, 17].

Forum theatre's “learning by doing” approach is particularly effective in health professions education because it strengthens clinically relevant competencies such as communication and empathy [18, 19, 20]. This participatory format encourages audience members to interrupt their observation and step into character, allowing them to actively engage with the scenario through embodied experience rather than passive observation [21]. Forum theatre involves playacting a scenario with a protagonist (e.g., an ED patient presenting with an EPL), an antagonist (e.g., a health care provider communicating without compassion or in an uninformed way), and bystanders (e.g., EM residents, faculty learners) witnessing the interaction, known as “spect‐actors” [22]. Forum theatre also includes a “joker,” who facilitates the experience by remaining non‐judgmental, promoting open dialogue among participants, and fostering a safe space for individuals to share their thoughts and experiences [21].

Forum theatre involves performing a scene, then repeating the same scene but inviting the spect‐actors to step into the role of one of the actors, affecting the direction and outcome of the interaction with the goal of provoking positive change (e.g., improving care and communication around EPL in an ED). Once a spect‐actor steps into one of the roles, the remaining actor immediately responds and engages with the new direction of the scene. The goal is to have multiple substitutions to explore different perspectives, approaches, and outcomes.

Forum theatre facilitates experiential learning through repeated practice of communication strategies, structured reflection, active audience participation, and exploration of diverse perspectives and responses to emotionally complex clinical encounters [21]. ED interactions with patients experiencing EPL require flexible, context‐sensitive communication that is responsive to patients' emotions, values, and evolving needs rather than reliance on a scripted response [22, 23]. This technique is particularly effective for teaching compassionate PCC because learners can rehearse, evaluate, and revise communication strategies through repeated enactment and reflection unlike traditional simulation or role‐play, which often focus on demonstrating competence during a single interaction [18, 20, 22]. Forum theatre has been used in various health professions disciplines, such as medicine [24], midwifery [25], and nursing [18], but its application to training EM residents in compassionate communication techniques when caring for a patient experiencing EPL is unique and innovative.

## 3 Objective of Innovation

The intervention's goal was to answer Gregory et al.'s call to address “an urgent need for improved education for ED healthcare professionals on … empathetic communication … and trauma‐informed approaches tailored to reproductive loss” [5]. To do this, we created a novel, experiential learning exercise using forum theatre techniques to help EM residents observe, practice, and refine their communication skills with patients experiencing EPL.

## 4 Development Process

We are a multidisciplinary team of communication scholars, EM physicians, simulation faculty, and arts‐based researchers who developed this EPL training experience. To enhance credibility, content experts reviewed the case, script, and learning objectives to ensure authenticity, educational relevance, and alignment with PCC principles. We developed a 40‐min forum theatre exercise for post‐graduate EM residents that was implemented into a regularly scheduled simulation training session. The forum theatre portion featured an EPL case depicting ineffective patient‐provider communication. The case was informed by our (K.E.B., M.B., and J.J.B.) two decades of research on communication about reproductive loss, including EPL, and reviewed by two EM physicians (A.B.A. and M.J.M.) for clinical accuracy and realism. Rooted in trauma‐informed compassionate communication techniques [26, 27, 28], we intentionally wrote the case to violate the three patient‐centered care principles—recognition (i.e., allow space to reveal distress), exploration (i.e., respond to emotions), and therapeutic action (i.e., communicate to address distress)—outlined in Brann et al.'s [28] communication model, which is grounded in the context of reproductive loss. For example, the physician dismisses the patient's uncertainty (i.e., recognition), overlooks emotional concerns (i.e., exploration), and neglects offering supportive resources (i.e., therapeutic action). Through a dramatized EPL scenario that models poor communication, forum theatre gives learners the opportunity to practice delivering bad news and responding to emotional cues.

## 5 The Implementation Phase

This study was approved by Indiana University's Institutional Review Board and conducted at a simulation center associated with a large urban academic medical center. We administered the training twice with two different groups of learners (*N* = 31). The first training included eight EM postgraduate year (PGY)‐2 residents who were divided into three groups (i.e., two groups of three residents and one group of two residents) during a regularly scheduled, self‐selected didactic. The second training included 23 EM PGY‐1 residents divided into four groups (i.e., three groups of six residents and one group of five residents) during a non‐clinical orientation month before beginning residency. All residents were verbally notified that video data would be collected for educational and research purposes and that participation in the research was voluntary.

Residents began the training with a simulation modeled after their typical didactic sessions. They entered a room in a simulated ED environment with the task of assessing a female patient presenting with abdominal pain from an unknown etiology, who expresses concern for EPL. The patient was portrayed by a high fidelity Laerdal 3G Sim Man manikin with female voice, long hair, and patient gown. The voice was provided by a female simulation technician with scripted responses. A faculty embedded participant (M.J.M.) performed the nurse role. The residents were provided bedside ultrasound images and clinical presentation that supported the diagnosis of missed abortion and EPL. The residents had up to 10 min to make a diagnosis and communicate their findings to the patient. After this time, during the first training session with PGY‐2 residents, faculty research team member A.B.A. entered the room, and M.J.M. and A.B.A. led a structured debrief of the clinical assessment using the PEARLS framework for simulation debriefing [29]. The PEARLS debriefing framework guides learner reflections through four phases: reactions (e.g., How are you feeling about that experience?), description (e.g., What were the main things that needed addressed?), analysis (e.g., What aspects of the case do you think were handled well? What were challenging?), and summary (e.g., What is one take‐away you can implement in your own practice?) [29]. After the debrief, M.B., J.J.B., and E.G.R. introduced and outlined the forum theatre exercise, which was new to all residents.

After confirming that the residents correctly diagnosed the patient, M.B. explained that they would observe an interaction based on an actual physician‐patient encounter, in which an actor portraying a physician (E.G.R.) delivered the diagnosis and discussed next steps with a patient (J.J.B.). After watching the scene, the joker, M.B., restarted the scene from the beginning, inviting residents to say “stop” at any point when they witnessed something that they wanted to do differently. They were then invited to step in and take an actor's role (physician or patient) to alter the interaction. M.B. then resumed the scene from that moment, continuing to encourage participation throughout. If the scenario continued for more than 1 min without intervention, M.B. paused the scene and asked the audience to suggest alternative communication approaches, ensuring all residents participated as spect‐actors. After multiple replays of the scene, M.B. facilitated an interactive debrief using the PEARLS framework [29] while the other authors shared observations about residents' communication behaviors. Before leaving the simulation, residents received a one‐page handout describing evidence‐based compassionate communication practices and a QR code linking to a survey. The survey included six Likert‐type questions (0 = *not at all*; 4 = *a great deal*) assessing the forum theatre exercise's effect on empathy, confidence, and understanding of effective communication during EPL care. It also included two open‐ended questions about participants' key takeaways and final comments, followed by five demographic items. All learners completed the optional post‐survey.

The second training session with PGY‐1 residents followed the same format, with two notable exceptions. First, because the EM PGY‐1 residents were beginning their residency and had limited clinical experience, they worked in pairs to determine the diagnosis and communicate it to the patient during the simulation. Second, only three authors were present to lead the training. M.J.M. served as both the nurse during the simulation and the physician in the forum theatre exercise. J.J.B. portrayed the patient, and M.B. served as the joker and facilitated the debrief. The patient‐provider script and actor instructions, along with the learner handout, are available as supplemental material accompanying the online article.

## 6 Outcomes

Thirty‐one EM residents participated in the forum theatre training, including 20 men (i.e., 13 PGY‐1; 7 PGY‐2) and 11 women (i.e., 10 PGY‐1; 1 PGY‐2). Participants had an average age of 28.77 years (SD = 3.00) and graduated medical school between 2020 and 2026 (64.5% in 2026).

Overall, the experience was positively received, with mean scores exceeding 3.00 on the 4‐point Likert‐type scale for all survey items (see Table 1 for item response distributions, means, and standard deviations). The highest‐rated item indicated that the forum theatre exercise provided practical examples of how to communicate compassionately with pregnant patients experiencing EPL in the ED (*M* = 3.39, SD = 0.88). Additionally, participants agreed that the training enhanced their empathy (*M* = 3.13, SD = 0.96) and confidence in communicating compassionately (*M* = 3.03, SD = 0.75), helped them recognize the importance of addressing patients' emotional needs (*M* = 3.23, SD = 0.92), and encouraged reflection of their own communication behaviors (*M* = 3.29, SD = 0.97).

**TABLE 1:** Item distributions, means, and standard deviations of survey responses.

Question	*Not at all*, *n (%)*	*A little*, *n (%)*	*A moderate amount*, *n (%)*	*A lot*, *n (%)*	*A great deal*, *n (%)*	*M* (SD)
How much did the forum theatre activity enhance your ability to empathize with patients who experience EPL in the ER?	1 (3.2)	0 (0.0)	6 (19.4)	11 (35.5)	13 (41.9)	3.13 (0.96)
How effectively do you think the forum theatre activity illustrated the impact of compassionate communication on patients who have experienced EPL?	1 (3.2)	0 (0.0)	4 (12.9)	13 (41.9)	13 (41.9)	3.19 (0.91)
How confident do you feel in applying compassionate communication techniques demonstrated in the forum theatre activity in your professional practice?	0 (0.0)	0 (0.0)	8 (25.8)	14 (45.2)	9 (29.0)	3.03 (0.75)
To what extent did the forum theatre activity help you recognize the importance of addressing emotional needs in EPL care?	1 (3.2)	0 (0.0)	4 (12.9)	12 (38.7)	14 (45.2)	3.23 (0.92)
To what extent did the forum theatre activity inspire you to reflect on and improve your own communication practices?	1 (3.2)	0 (0.0)	5 (16.1)	8 (25.8)	17 (54.8)	3.29 (0.97)
To what extent did the forum theatre activity provide practical examples of how to communicate compassionately with pregnant patients who have experienced EPL in the ER?	1 (3.2)	0 (0.0)	2 (6.5)	11 (35.5)	17 (54.8)	3.39 (0.88)

*Note:* Assessed on a 5‐point Likert scale ranging from 0 to 4: 0 = *Not at all*, 1 = *A little*, 2 = *A moderate amount*, 3 = *A lot*, and 4 = *A great deal*.

Nearly all participants expressed immediate acceptance of forum theatre, as reflected in their responses on the post‐survey: “Really liked being forced to step in to take over the scene” (PGY‐2), “Thought it was an interesting way to practice scenarios and see different approaches to same one” (PGY‐2), “I liked starting with a scene of less ideal communication to already start formulating what I would do differently” (PGY‐2), and “This was a very valuable experience for me and will help my future patients!” (PGY‐1). It is encouraging that learners recognized “That you can always improve; there are multiple correct ways to deliver different types of news and information and communicate” (PGY‐2), “There is not a single formula to effectively delivering this news, but there are several principals to follow” (PGY‐1), “Small changes in conversation can have a big impact on the process of emotional processing” (PGY‐1), and that “Practice helps” (PGY‐2) when developing skills to effectively communicate with patients experiencing EPL. When sharing what they learned, participants commented, “How to discuss hard topics with patients in a compassionate way” (PGY‐1), “Language is super important. Watch exactly what you are saying and avoid technical jargon at all costs. Avoid any terminology that could be misconstrued” (PGY‐1), “The value of mirroring patient language” (PGY‐1), and “Correct and respectful terminology, patient‐based approach to preferred language” (PGY‐2). Some even shared specific examples of language they learned to avoid: “Using word abortion” (PGY‐2), “Phrasing alternatives to products of conception/tissue” (PGY‐1), “Use other words instead of ‘bad news’” (PGY‐2), “Patients don't like the word common” (PGY‐1), or “Women don't want to hear stats of that this is a normal experience” (PGY‐2).

Some participants shared that they learned “How to prime the patient for bad news” (PGY‐1), and learners recognized the value of both verbal and nonverbal communication. They mentioned the “Importance of pauses and silence in these discussions” (PGY‐1) for “Giving space for the patient to process” (PGY‐2) or just remembering to “Give space … [and] offer resources” (PGY‐2). Other comments about nonverbal behaviors included: “Don't forget to sit down” (PGY‐1) and “Being mindful of the words I use and the nonverbal communication is the next big step for me in developing this skill” (PGY‐1). Together, these responses suggest that the experience not only enhanced participants' awareness of compassionate, patient‐centered language but also increased their confidence in tailoring communication, avoiding harmful phrasing, and supporting patients' emotional processing during EPL in the ED.

## 7 Reflective Discussion

Recognizing the need for compassionate communication training in the ED, particularly for pregnancy loss care [5], we developed and implemented an experiential learning exercise within a scheduled simulation encounter. Although residents responded positively and reported empathy, confidence, and awareness of patients' emotional needs, these findings reflect a Kirkpatrick Level 1 (Reaction) evaluation [30] and are limited by the small sample size from a single site and the use of an immediate post‐training self‐report assessment that measured participants' perceptions rather than knowledge retention, behavior change, or patient outcomes. Nevertheless, evaluating learners' reactions and perceived learning is appropriate for educational interventions designed to support professional development [30], and future longitudinal evaluation is warranted to assess the transfer of communication behaviors to clinical practice and their effects on patient outcomes.

Even with these limitations, forum theatre appears to be a valuable tool that could be adapted for other post‐graduate training and/or clinical situations requiring ED providers to deliver bad news, which was recognized by the residents as well. For example, a resident commented learning “How to be clear and concise when delivering news, not only about pregnancies but other bad news” (PGY‐1). Additionally, in follow‐up communication with one of the participants 6 weeks after the training, one PGY‐2 resident shared that he reflected on this experience and used the techniques he learned when “breaking news of likely cancer to a patient.”

Based on participant feedback, observations from educators, and our own analyses of the interactions, this experience shows strong promise for modeling compassionate communication and providing learners with meaningful opportunities to practice these skills. We view this approach as both sustainable and adaptable, and plan to continue using forum theatre as a learning modality across additional simulated cases. This approach is not time intensive nor does it require many resources; thus, it can be embedded into existing curricula to demonstrate and practice key communication skills. For example, faculty volunteers served as actors in the forum theatre exercise, reducing the time and cost associated with standardized patients. Additionally, because the focus is on communication, the forum theatre portion of the training does not require specialized medical or simulation equipment.

When implementing forum theatre into health care training, we advise demonstrating the roles of actors and spect‐actors with educator clinicians, rather than only describing the process, to help learners feel more comfortable interrupting and engaging with their colleagues. After seeing a delay in participants' willingness to intervene during the first interaction of the first training, two authors (M.J.M. and A.B.A.) paused the scene at different times to demonstrate how to interject and alter the scene. This appeared to increase participants' comfort as all learners ultimately engaged and participated in the experience. Additionally, it is important to be flexible with forum theatre. Traditionally, the joker does not interject, instead facilitating the interaction as it unfolds; however, due to time constraints in the simulation, M.B. paused the scenario if no one intervened within 1 min to encourage participation. This adjustment did not appear to negatively affect the outcomes, and instead, may have enhanced engagement as more participants actively contributed. In fact, the authors would consider prompting an interruption at even more frequent intervals (e.g., 30 s) depending on the overall time allotment to maximize participation.

This training experience suggests that forum theatre is a promising modality for teaching compassionate communication to EM residents. Residents reported that having the opportunity to observe and practice communication behaviors in a psychologically safe environment enhanced empathy and confidence for communicating with a patient experiencing EPL in an emergency setting. Based on positive feedback from both PGY‐1 and PGY‐2 residents, we recommend forum theatre as an innovative educational approach for addressing communication training gaps in residency education.

## Author Contributions

**Maria Brann:** conceptualization, methodology, investigation, formal analysis, writing – original draft, writing – review and editing. **Jennifer J. Bute:** conceptualization, methodology, investigation, writing – review and editing. **Kelsey E. Binion:** conceptualization, methodology, writing – review and editing. **Emilio G. Robles:** conceptualization, methodology, writing – review and editing, investigation. **Andreia B. Alexander:** conceptualization, methodology, investigation, writing – review and editing, project administration. **Malia J. Moore:** methodology, investigation, writing – review and editing, project administration.

## Conflicts of Interest

The authors declare no conflicts of interest.

## Supporting information


**Data S1:** Supporting Information.

**Data S2:** Supporting Information.

**Data S3:** Supporting Information.

## Data Availability

The data that support the findings of this study are available from the corresponding author upon reasonable request.
