# Supplementary material for: Compassionate Communication During Early Pregnancy Loss: Using Forum Theatre to Train Emergency Medicine Residents
Source: AEM Educ Train. 2026 Sep 27;10(5):e70260. doi: 10.1002/aet2.70260 (PMC13616817; doi:10.1002/aet2.70260)
Supplement: Supplementary file 1 [file AET2-10-e70260-s001.pdf]

Early Pregnancy Loss Emergency Department Encounter Forum Theatre Script

Setting: Emergency Department, curtained-off bay, busy night.

Patient: Jane Smith is in her first trimester of pregnancy when she began cramping. Unsure of what was occurring and whether she should be concerned, she went to the Emergency Department to be examined. She is sitting on the bed, visibly anxious, waiting for the physician after completing lab work.

Dr. Morgan enters, glancing at a chart (either clipboard or tablet) in their hands.

Dr. Morgan:
Jane? I'm Dr. Morgan. I've reviewed your labs and chart and (brief pause while looking at chart) ... it looks like you're having a spontaneous abortion.

Jane:
(with a confused, quick, and louder voice) Wait—what? What does that mean?

Dr. Morgan:
It means the pregnancy isn't viable. There's no fetal heartbeat. It's a miscarriage.

Jane:
(slowing down, concerned look on face) Oh… I didn't know. I thought everything was okay. I was just having some cramping. I was still feeling sick and everything.

Dr. Morgan:
Yeah, symptoms can be misleading. These things happen. It's actually really common—about one in five pregnancies ends this way. Nothing to be alarmed about.

Jane:
But (pause) … I don't understand. Is there something I did wrong?

Dr. Morgan:
No, it's usually chromosomal. Not preventable. It's just biology. Your body recognized something wasn't right and ended the pregnancy.

Jane:
(quietly) I didn't even get to hear the heart...

Dr. Morgan:
(interrupting) Right. So, we need to talk about next steps. You can either wait for the tissue to pass naturally, take medication to speed it up, or have a minor procedure called a D&C. We don't do that here, but we can refer you.

Jane:
(hesitant) I… I don't know what to do. (overwhelmed) This is a lot.

Dr. Morgan:
Well, I'll have the nurse bring you a pamphlet with the options, but you'll need to decide soon. We can't keep you here much longer. There's not much we can do and we're very busy tonight.

Jane:
(questioning) Can I talk to someone about this? Like… a counselor or something?

Dr. Morgan:
We don't really have that in the ED. You can follow up with your OB or maybe a therapist if you have one.

Jane:
(weary) I just feel really alone right now.

Dr. Morgan:
Yeah, I get that. But unfortunately, this isn't something we can fix here. You'll want to follow up with outpatient care. I'll go ahead and put in the discharge orders.

Jane:
Okay…

Dr. Morgan:
Alright. The nurse will be in shortly. Take care.

Dr. Morgan exits without further acknowledgment.

*****

Actor Instructions

During re-enactment, if "spect-actor" enters the scene as the physician, the patient should continue to ask questions, express confusion and uncertainty both verbally and nonverbally, and repeat concerns if not addressed by the physician.

If "spect-actor" enters the scene as the patient, the physician should continue to use short responses and focus on discharging the patient quickly, use a neutral tone, provide only factual information, and focus on physical health, not emotional concerns.
