# Supplementary material for: Compassionate Communication During Early Pregnancy Loss: Using Forum Theatre to Train Emergency Medicine Residents
Source: AEM Educ Train. 2026 Sep 27;10(5):e70260. doi: 10.1002/aet2.70260 (PMC13616817; doi:10.1002/aet2.70260)
Supplement: Supplementary file 2 [file AET2-10-e70260-s002.pdf]

Supplementary Index

Learner_Handout.pdf
Handout describing patient-centered care principles and compassionate communication examples. (PDF, 1 page, 152 KB)

Patient_Provider_Script_Actor_Instructions.pdf
Early Pregnancy Loss Emergency Department encounter forum theatre script for patient and provider characters and actor instructions. (PDF, 2 pages, 124 KB)
