# Supplementary material for: Compassionate Communication During Early Pregnancy Loss: Using Forum Theatre to Train Emergency Medicine Residents
Source: AEM Educ Train. 2026 Sep 27;10(5):e70260. doi: 10.1002/aet2.70260 (PMC13616817; doi:10.1002/aet2.70260)
Supplement: Supplementary file 3 [file AET2-10-e70260-s003.pdf]

# COMPASSIONATE COMMUNICATION DURING PREGNANCY LOSS

Patient-centered care and compassionate communication during perinatal loss help patients understand their experience and plan for recovery and the future. The communication strategies below reflect patient-centered approaches that align patient's needs, perspectives, and values after pregnancy loss.

| Patient-Centered Care Principle | Compassionate Care Behavior | Communicative Examples | Language Examples |
|---|---|---|---|
| **Recognition**<br><br>Creating and allowing space for patients to reveal emotional distress | **Demonstrate empathy** | Respond to patient nonverbal and verbal cues | "How much detail should I share to make my response helpful for you?" |
| | | Provide patient with full attention | "Please tell me more." |
| | | Provide comfort and reassurance | "You are not alone." |
| | | Acknowledge the loss | "I am so sorry about your loss." |
| | | Validate emotions | "It's common to feel a mix of emotions after a loss like this, and all those feelings are valid." |
| | **Provide space for patients to process** | Remain silent as the patient shares | |
| | | Avoid rushing the conversation and/or patient | "Take all the time you need." |
| | | Be responsive to questions | "That's a good question." |
| | **Employ facilitative communication** | Use "we" language | "We will get through this together, and I will explain each step, so you feel supported." |
| | | Listen actively | "Mm hmm." |
| | | Establish rapport by identifying a connection | "I can only imagine how difficult this may be." |
| **Exploration**<br><br>Responding to emotions | **Check for understanding** | Ask open-ended questions | "How are you feeling?" |
| | | Use the teach-back method | "To be sure we are on the same page, how would you put what I said in your own words?" |
| | **Be sensitive to patient knowledge and preferred language** | Avoid clinical terms ("spontaneous abortion," "fetal demise") and emotionally charged language | "This means the pregnancy is no longer continuing." |
| | | Avoid overly detailed descriptions of procedures and/or graphic details | "You will likely experience intense cramps and bleeding." |
| **Therapeutic Action**<br><br>Communicating to address emotional distress | **Offer support** | Provide information (e.g., additional tests) | "Your OB/GYN may recommend tests like blood work or an ultrasound to help understand the cause of your loss and guide next steps." |
| | | Provide resources (e.g., support groups, referrals) | "Let me share with you some pregnancy loss support groups in the area that you may find helpful." |


Brann, M., Bute, J. J., & Scott, S. F. (2020). Qualitative assessment of bad news delivery practices during miscarriage diagnosis. *Qualitative Health Research, 30*(2), 258-267. https://doi.org/10.1177/1049732319874038

Brann, M., Bute, J. J., & Binion, K. (2025). "It's not an intervention; it's a philosophy": Extending a model of patient-centered communication to pregnancy after loss. *Health Communication, 40*(14), 3008-3020. https://doi.org/10.1080/10410236.2025.2489177
